# 髓系相关基因突变的变异等位基因频率在骨髓增生异常综合征研究中的应用

**DOI:** 10.3760/cma.j.cn121090-20240806-00293

**Published:** 2025-04

**Authors:** 铭玥 尚, 力 苏

**Affiliations:** 首都医科大学宣武医院血液内科，北京 100032 Department of Hematology, Xuanwu Hospital, Capital Medical University, Beijing 100032, China

## Abstract

骨髓增生异常综合征（Myelodysplastic syndrome，MDS）是一种异质性髓系克隆性恶性肿瘤，患者预后差异较大。研究发现多种髓系相关基因突变与MDS的诊断及预后相关，因此MDS预后国际工作组（IWG-PM）及其他预后评估体系中均加入了多种髓系相关基因突变作为MDS诊断及预后依据。应用变异等位基因频率（Variant allele frequency，VAF）检测技术进一步研究发现，髓系相关基因在MDS的病程中发生动态演变，并呈现一定的规律性。本文对髓系相关基因突变VAF在MDS研究中的应用进行概述，强调基因突变VAF在MDS分型、危险分层和治疗反应等方面的重要价值。

## Abstract (en)

Myelodysplastic syndrome（MDS）is a heterogeneous clonal myeloid malignancy with a variable prognosis. Some myeloid-related gene alterations have been found to be associated with the diagnosis and prognosis of MDS. As a result, multiple myeloid-related gene mutations have been added to International Working Group for Prognosis in MDS（IWG-PM）and other MDS prognosis evaluation systems as diagnostic and prognostic indications. Further research using the variable allele frequency（VAF）detection technique demonstrated that myeloid-related genes evolve dynamically during the course of MDS with some regularity. Furthermore, significant relevance have been found between the VAF evolution of particular myeloid-related genes and MDS subtypes, risk classification, and prognosis. This article provides an overview of the application of VAF of myeloid-related gene mutations in MDS research, highlighting the critical role of gene mutation VAF in MDS subtype categorization, risk stratification, and therapy response assessment.

骨髓增生异常综合征（Myelodysplastic syndrome，MDS）是一种异质性髓系克隆性恶性肿瘤，以外周血细胞减少、骨髓发育异常及造血功能低下为临床特点，同时其高风险向急性髓系白血病（Acute myeloid leukemia，AML）转化的特征使MDS患者表现出不同的预后，预期寿命从几个月至多年不等[1]。

目前以2016版世界卫生组织（WHO）MDS分型诊断和MDS国际预后评分系统（IPSS）及其修订版（IPSS-R）预后分层标准的应用最为广泛，均将患者骨髓原始细胞比例、外周血细胞减少和有明确预后意义的细胞遗传学异常作为预后评估的主要依据。然而，细胞形态学的诊断结果依赖诊断者的主观判断，影响MDS诊断的准确性。

应用第二代基因测序（NGS）技术发现超过50种MDS相关的髓系基因突变及其临床应用价值，如RUNX1、SF3B1和NRAS等基因突变与严重血小板减少症或骨髓造血干细胞比例增加等临床表现密切相关[2]，TP53、EZH2和ASXL1等多种基因突变状态有独立的预后意义[2]–[4]。SF3B1突变与环状铁粒幼细胞有强相关性，双等位TP53（biTP53）突变可明确预示不良预后。在此基础上，2022年国际共识分类（ICC）[5]和WHO[6]均首次制定了以基因组特征定义的MDS亚型，即MDS-SF3B1和MDS-biTP53。同年MDS预后国际工作组（IWG-PM）构建了IPSS-Molecular（IPSS-M）预后评估工具，该评分在IPSS-R预后分层的基础上加入了31个预后相关基因突变作为分层依据，更客观、系统地判别MDS患者的临床预后。

MDS病程中除基因突变可能发生动态演变外，同一基因的变异等位基因频率（Variant allele frequency，VAF）也可能不断改变且呈现一定的规律性。目前的研究已证实某些基因突变状态（是否突变）与MDS的分型、危险分层及预后有显著相关性，但其VAF的变化与MDS患者的临床特征、治疗反应或预后的相关性有待进一步验证。本文将对髓系相关基因突变的VAF在MDS研究中的应用进行概述。

一、单个或多种髓系相关基因的VAF与MDS

VAF是指支持特定变异等位基因的测序reads数占基因组位点内reads数总数的比例，是衡量携带特定基因组变异的等位基因比例的指标[7]–[8]，计算公式为VAF（％）＝突变reads数/（突变reads数+野生reads数）×100％。基于NGS的VAF检测方法包括全基因组测序（WGS）、全外显子组测序（WES）和靶向测序等，可在不同测序深度下提供不同的基因组覆盖范围[9]。大多数MDS相关髓系突变基因VAF的临床相关性尚不明确，以下所列举的髓系相关基因的研究较为集中。

1. TP53：TP53被普遍认为是一种预后不良的生物标志物，也是MDS突变频率较高的基因之一。Sallman等[10]对219例MDS或继发性AML患者的20余种突变基因VAF进行分析，发现TP53 VAF>40％的MDS患者的中位总生存（OS）期为124 d，而VAF<20％的患者中位OS期未达到（*HR*＝3.52，*P*＝0.01），同时多因素Cox分析表明TP53突变状态和TP53 VAF>40％具有预后意义（*HR*＝1.47，95％ *CI*: 1.16～1.86，*P*＝0.002；*HR*＝1.61，95％ *CI*: 1.25～2.08，*P*<0.0001）。随后Belickova等[11]在154例低危MDS患者TP53 VAF的研究中通过OS的评估确定了以6％作为患者预后分层的TP53 VAF最佳临界值。Montalban-Bravo等[12]以20％～50％作为VAF的临界值对TP53突变的MDS患者进行分组，三组患者生存情况的分析结果再次证实了高TP53 VAF的MDS患者预后更差。因此，多项研究一致认为，TP53 VAF应独立于临床预后评分系统划分不同的预后组[10]–[13]。此外，一项包括11项研究、共计4 003例MDS患者的荟萃分析为TP53 VAF临界值的确定提供了参考，结果表明20％的临界值是高低克隆负荷之间的粗略界限，以40％作为临界值指导治疗较为精确，且TP53 VAF>40％是影响MDS患者OS的独立不良预后因素[14]。

需要注意的是，7％～11％的MDS患者可检测到多种类型的致病性TP53突变，其中约三分之二为biTP53突变[15]–[16]，且单/双等位TP53突变对预后的影响截然不同。一项纳入3 324例MDS患者的队列研究[15]表明，单等位TP53突变患者与野生型患者相比，VAF>23％的患者死亡风险增加（*P*<0.001），而VAF≤23％患者的OS与野生型患者相似；biTP53突变患者在所有VAF范围内的预后都很差。因biTP53突变有较明确的预后意义，2022年WHO发表的MDS分型正式将“MDS-biTP53”列为新的亚型[6]。

2. SF3B1：在MDS中，SF3B1突变决定了以存在骨髓环状铁粒幼细胞为特征的疾病状态，IWG-PM提出，SF3B1突变型MDS应作为一种独特的疾病亚型[17]。目前大部分研究认为SF3B1突变与MDS患者生存率高有关[4],[18]–[21]，但其对立观点也不容忽视[2],[22]–[24]，如一项纳入9个队列、共2 259例MDS患者的荟萃分析表明，SF3B1突变与MDS患者的OS无关[24]。但在讨论SF3B1 VAF水平与OS之间的关系时得到了不同的结论，即多因素回归分析表明，高VAF（≥15％）与良好预后呈独立相关（*HR*＝0.52，*P*＝0.048），且与低VAF（<15％）MDS患者相比，高VAF患者多属于MDS伴环状铁粒幼细胞（MDS-RS）亚型（*P*＝0.012）[21]。也有研究发现，SF3B1 VAF与骨髓环状铁粒幼细胞比例（*P*＝0.002）或骨髓幼红细胞比例（*P*＝0.01）相关[10],[25]。

3. U2AF1：目前关于U2AF1突变负荷对预后影响的报道相对较少。一项纳入234例MDS患者的研究进行了初期探索，若以U2AF1 VAF中位数（39.5％）为临界值，与低VAF组（VAF≤40％）相比，高VAF组（VAF>40％）的1年OS率降低（46.1％对80.5％，*P*＝0.027），并且7号染色体变异的发生率也明显更低（46.1％对80.5％，*P*＝0.027），而两组患者在治疗反应、疾病进展和复发方面无明显差异，并通过多因素Cox分析首次证实了U2AF1 VAF>40％是MDS患者短OS期的独立因素[26]。同时，U2AF1与TET2、ASXL1和RUNX1发生共突变时，进一步验证了U2AF1突变负荷对疾病进展和预后的影响[26]。此外，近年的研究发现不同的U2AF1突变位点在患者预后上存在差异，如Q157位点突变的患者与S34位点突变相比预后更差[27]–[28]，因此今后的研究可能还需结合突变位点来探讨U2AF1 VAF的临床意义。

4. TET2：有研究分析了159例低风险MDS患者的15个MDS相关突变基因，发现TET2 VAF≥18％的患者OS期明显短于TET2 VAF<18％或无突变的患者（中位OS期：20.4个月对47.8个月，*P*＝0.020），并将TET2 VAF≥18％作为独立因素纳入新的预后评分系统，进一步将低风险MDS患者分为3个预后不同的组（*P*<0.001）[29]，进而揭示了TET2突变负荷与预后的关联。另有研究分析了382例新诊断MDS患者基因突变谱发现，在多因素Cox分析中，TET2突变状态本身与OS无关（*HR*＝1.32，95％ *CI*: 0.86～2.04，*P*＝0.209），而高TET2 VAF（≥32％）是不良预后的独立预测因素（*HR*＝1.69，95％ *CI*: 1.07～2.69，*P*＝0.025）[21]。

5. 多种髓系相关基因的VAF与MDS：Lee等[30]对698例MDS患者中与骨髓恶性肿瘤相关的突变VAF进行了全面分析，单因素Cox回归分析证明DNMT3A、ASXL1、EZH2、SETBP1、BCOR、SFSF2、ZRSR2突变的高VAF与患者预后相关，并且在多因素分析中证明，具有高VAF的DNMT3A（>40％）和ZRSR2（>60％）突变是影响OS的独立不良预后因素。这些发现强调了将VAF纳入MDS危险分层和治疗决策的潜在重要性。但并未充分得出多种髓系相关基因突变的VAF与患者预后的具体关系。这可能需要更多的样本量和足够的临床观察时间才能得出相关结论。

目前关于高VAF导致显著临床效应的详细机制仍不清楚，但大多认为由于VAF代表克隆负荷，克隆负荷足够大才能对患者生存产生显著影响。另外，当前的研究仍存在局限性：VAF并不是影响突变的临床和预后影响的唯一因素，其他突变特征包括突变位点、突变类型（错义突变等）和共突变基因也可能发挥重要作用[31]–[32]，未来需要更多大规模的研究来证实当前的发现。

二、髓系相关基因突变的VAF值变化揭示MDS的演变规律

目前研究认为在造血干细胞不断分裂和分化过程中，基因突变会随机发生，当基因突变发生在基因组编码区，会影响细胞自我更新和分化功能，从而使细胞获得选择性生长优势，直接导致癌症发生和发展，这类突变称为驱动突变[33]，这可能对疾病的发病机制研究至关重要。

1. 驱动突变VAF揭示MDS基因突变的发生顺序：目前已发现超过30种驱动突变参与MDS的发病过程[3]–[4]，这些驱动基因可被分为不同的功能途径，包括DNA甲基化、RNA剪接、染色质修饰和信号转导等。携带驱动突变的细胞进行克隆性增殖，进而累积获得相对更高的驱动突变VAF[34]，揭示MDS病程中基因突变的演变规律。研究发现，参与DNA甲基化（DNMT3A、TET2、IDH1和IDH2等）和RNA剪接（如SF3B1、SRSF2、U2AF1和ZRSR2等）的基因突变往往表现出更高的VAF，表明这些突变的起源更早，而参与染色质修饰（EZH2和ASXL1等）和信号传导（RUNX1、GATA2和BCOR等）的基因驱动突变往往发生较晚[3]–[4]。相似的规律也存在于继发性AML（sAML）患者中，约30％的MDS患者最终进展为sAML，遗传学研究证明两者基因突变谱具有相似性，如DNMT3A、TET2、ASXL1和剪接基因等，这证实两者是一个疾病的连续体。同时两者基因突变谱VAF的差异表明，疾病进展可能与克隆演变有关。

首先，Makishima等[35]通过对2 250例低危、高危MDS及sAML患者进行基因组测序发现，与高危MDS患者相比，sAML患者中Ⅰ型基因显著富集，即FLT3、NPM1、NRAS、PTPN11、WT1、IDH1、IDH2，而Ⅱ型基因在高危MDS中突变频率更高，如GATA2、RUNX1、TET2、ZRSR2、TP53、STAG2、ASXL1。同时对连续收集的样本进行分析发现，Ⅰ型突变往往为新获得突变而非丢失突变且其中位VAF明显低于Ⅱ型突变，最终得出结论：Ⅰ型突变在MDS向AML转化过程中起关键作用[35]–[36]。然而，目前对突变获得顺序的理解仍不完整，因为大部分数据是基于未配对的MDS和sAML患者队列中的基因突变频率，而不是来自同一患者的序列样本。

针对这一局限性，Menssen等[37]收集了60例具有配对序列的MDS/sAML样本的患者，发现在sAML中检测到的潜能未定的克隆性造血（CHIP）相关基因突变（如TP53、DNA甲基化和RNA剪接相关基因）大多以相对更高的平均VAF（分别为33％、43％和31％）存在于MDS样本中，这再次表明它们是在MDS病程早期获得的，而在sAML中较少检测到信号转导相关基因突变，再次验证了上述研究结果。

2. VAF揭示MDS克隆进化模式：通过在MDS患者疾病进程中进行多次基因VAF检测，多项研究发现了2种不同的克隆进化模式[35],[38]–[39]，包括线性进化（即在优势克隆的基础上递次出现新的亚克隆并逐渐扩增占据优势）和分支进化（即多种新克隆与原克隆共同扩增），同时在进化过程中常伴随着“克隆清扫”现象（即亚克隆逐渐扩增直至将其他克隆“清除”）[35]。克隆进化模式的发现为MDS的遗传驱动因素提供重要信息，相比于通过骨髓原始细胞计数来判断疾病进程，监测肿瘤负荷和克隆进化或能更早地提示疾病进展。

3. MDS前体状态的定义：临床发现在没有MDS骨髓形态学或细胞遗传学证据的个体中可检测到多种MDS相关基因突变，长期的随访观察验证了其对血液恶性肿瘤的倾向性，因此定义为CHIP和意义不明的克隆性血细胞减少（clonal cytopenia of unknown significance，CCUS）等，通过使用单分子分子倒位探针（smMIPs）检测到的突变中超过90％的VAF低于3.5％[40]，因此学者们对突变克隆的最小规模出现争议。最初，取2％为CHIP髓系肿瘤相关基因突变VAF的临界值，这是WES检测体细胞变异的下限值[41]，但或许不能完全代表该值的生物学和临床相关性。Young等[42]研究发现基因突变VAF>1％（与低VAF相比）会显著增加AML风险（*OR*＝5.4，95％*CI*:1.8～16.6，*P*＝0.003）。另一项研究对90例接受造血干细胞移植的成人MDS患者移植前和移植后30 d的样本进行分析，结果显示移植后VAF为0.5％与较高的疾病进展风险和无进展生存（PFS）期缩短有关[43]。因此，对于VAF低于2％的突变状态与临床的相关性仍有待定论，未来仍需大样本量的分析研究加强其说服力。

三、VAF揭示克隆演变与治疗的关系

1. 治疗影响克隆演变的模式：目前研究发现化疗和造血干细胞移植等治疗方式能够影响克隆进化的模式。del（5q）是MDS和sAML中最常见的染色体异常之一，这部分患者经来那度胺治疗后，异常的del（5q）克隆可被抑制至极低水平，但伴随着疾病的进展或复发，del（5q）可能重新出现并与新的突变（如TP53等）或遗传学异常迅速扩增[44]–[45]，同样的情况也出现在移植后。另外，治疗过程可导致亚克隆的清除，而优势克隆可不受治疗的干扰，当疾病进展或复发时，新的突变可能跟随原有的克隆一起出现[46]。这些发现均初步证明了治疗方式能够对MDS病程中的克隆进化产生影响。

2. VAF的高低预示治疗效果：DNA去甲基化药物（HMAs）是唯一一类被批准用于治疗高风险MDS的药物，TET2突变与MDS患者HMAs治疗反应的相关性不断得到证实[47]–[48]，有多项研究表明[21],[48]，较低TET2 VAF而言，高TET2 VAF患者对HMAs的反应增加，其中Bejar等[48]研究指出TET2 VAF>10％预示着对HMAs反应率将提高，尤其在无ASXL1突变的MDS患者中疗效更加明显。另外，Montalban-Bravo等[12]发现，与高VAF相比，TP53突变的低VAF有更高的HMAs总应答率。但VAF是否能作为治疗效果的预测因素并未得到广泛证实，未来仍需继续更大规模的队列研究继续探索其规律。

四、总结

得益于基因测序技术的普及，目前对MDS驱动突变谱的认识日益完善和成熟，除了基因的突变状态，突变VAF也可能有重要的临床意义。目前的研究已初步证明了TP53、SF3B1和TET2等突变的VAF与患者预后相关，这提示我们，需利用新一代测序数据来增强基于形态学的肿瘤负荷临床评估，这将有助于我们更准确地预测疾病进程和选择治疗时机，改善MDS患者的预后。

在未来的研究中仍有大量问题等待完善和解决。首先，在突变VAF检测技术方面，鉴于各种测序方法的优点和局限性，很难形成统一的临床使用标准，且不同的测序平台存在仪器测量等差异，导致显著的分析偏差和不正确的临床解释，对于突变VAF精确临界值的确定尤为关键；第二，某种突变VAF的高低可能受到多种外界因素的干扰而发生动态改变，且不同基因之间的相互作用也将对单个基因VAF作用的研究造成阻碍；第三，对于克隆的演变及VAF与预后关系的研究，缺少对同一患者的序列样本进行长期、多次的检测和收集分析，将影响我们对克隆演变和VAF的动态观察。未来仍需多中心、更大样本量的前瞻性临床试验进行验证。
